# *In Vitro* Antioxidant, Anti-Inflammatory, and Digestive Enzymes Inhibition Activities of Hydro-Ethanolic Leaf and Bark Extracts of *Psychotria densinervia* (K. Krause) Verdc

**DOI:** 10.1155/2022/8459943

**Published:** 2022-05-06

**Authors:** Jean Romuald Mba, Djamila Zouheira, Hadidjatou Dairou, Fanta S. A. Yadang, Nfor Njini Gael, Lawrence Ayong, Jules-Roger Kuiate, Gabriel A. Agbor

**Affiliations:** ^1^Centre for Research on Medicinal Plants and Traditional Medicine, Institute of Medical Research and Medicinal Plants Studies Cameroon, P.O. Box 13033, Yaoundé, Cameroon; ^2^Department of Biochemistry, University of Dschang Cameroon, P.O. Box 67, Dschang, Cameroon; ^3^Centre Pasteur Du Cameroun, Yaoundé, Cameroon

## Abstract

*Psychotria densinervia* hydro-ethanolic leaf extract (PHELE) and bark extract (PHEBE) were evaluated for antioxidant, anti-inflammatory, and inhibition of digestive enzymes activities. The antioxidant activity was characterized by 2,2-diphenyl-1-picrylhydrazyl (DPPH), 2,2′-azino-bis(3-ethylbenzothiazoline-6-sulfonic acid) (ABTS), ferric reducing antioxidant power (FRAP), total phenolic content (TPC), and total flavonoid content (TFC) assays. The anti-inflammatory activity was characterized by protein denaturation and antiproteinase tests, while the inhibition of the enzymes was assessed using *α*-amylase, *α*-glucosidase, lipase, and cholesterol esterase activities. PHELE presented low (*p* < 0.001) IC_50_ (59.09 ± 5.97 *μ*g/ml) for DPPH compared with ascorbic acid (71.78 ± 6.37 *μ*g/ml) and *PHEBE* (115.40 ± 1.21 *μ*g/ml). The IC_50_ of *PHELE* (262.4 ± 4.46 *μ*g/ml) and *PHEBE* (354.2 ± 1.97 *μ*g/ml) was higher (*p* < 0.001) than that of catechin (33.48 ± 2.02 *μ*g/ml) for ABTS. *PHELE* had high (*p* < 0.001) FRAP (341.73 ± 21.70 mg CE/g) than *PHEBE* (150.30 ± 0.32 mg CE/g). *PHELE* presented (*p* < 0.001) high TPC (270.05 ± 7.53 mg CE/g) and TFC (23.43 ± 0.032 mg CE/g) than *PHEBE* (TPC: 138.89 ± 0.91 and TFC: 20.06 ± 0.032 mg CE/g). *PHELE* showed antiprotein denaturation with IC_50_ (257.0 ± 7.51 *μ*g/ml) (*p* < 0.001) and antiproteinase activity (74.37 ± 1.10 *μ*g/ml) lower than *PHEBE* (316.1 ± 6.02 *μ*g/ml and 177.6 ± 0.50 *μ*g/ml), respectively. Orlistat inhibited lipase (*p* < 0.001) activity with IC_50_ (37.11 ± 4.39 *μ*g/ml) lower than *PHELE* and *PHEBE* (50.57 ± 2.89 *μ*g/ml and 62.88 ± 1.74 *μ*g/ml, respectively). *PHELE* inhibited cholesterol esterase with IC_50_ (34.75 ± 3.87 *μ*g/ml) lower than orlistat (54.61 ± 2.56) and *PHEBE* (80.14 ± 1.71 *μ*g/ml). *PHELE* inhibited *α*-amylase IC_50_ (6.07 ± 4.05 *μ*g/ml) lower than *PHEBE* (19.69 ± 6.27 *μ*g/ml) and acarbose (20.01 ± 2.84 *μ*g/ml). Acarbose inhibited *α*-glucosidase (*p* < 0.001) activity with IC_50_ (4.11 ± 3.47 *μ*g/ml) lower than *PHELE* (24.41 ± 2.84 *μ*g/ml) and *PHEBE* (38.81 ± 2.46 *μ*g/ml). *PHELE* presented better antioxidant, anti-inflammatory, and enzyme inhibition activity than PHEBE.

## 1. Introduction

Overweight and obesity are defined as abnormal or excessive fat accumulation, which presents a risk to health [1]. In 2016, more than 1.9 billion adults, 18 years and older, were overweight. Of these, over 650 million were obese [2]. Obesity is now at epidemic proportions globally, with more than 2.8 million people dying each year [3]. Obesity has been associated with the pathogenesis of chronic diseases, such as cardiac injury, hypertension, and type 2 diabetes [4]. Obesity and overweight are a consequence of lipid accumulation in the form of triglycerides in the adipose tissue [5]. The main source of adipocyte triglycerides is chylomicrons and very low-density lipoproteins (VLDL), which are often obtained from food or liver lipogenesis [5]. In the digestive system, the dietary lipids undergo hydrolysis catalyzed by pancreatic lipases, cholesterol esterase, and phospholipases to yield free fatty acids, free cholesterol, and lysophosphatidic acid, respectively. Carbohydrates will undergo hydrolysis catalyzed by salivary and pancreatic *α*-amylase as well as *α*-glucosidases to yield simple sugars. One of the approaches in the fight against obesity and related diseases such as type II diabetes would be to search for new molecules that are able to inhibit the activity of the digestive enzymes earlier mentioned. Secondary metabolites of certain plant extracts have shown their effectiveness in the fight against obesity by regulating oxidative stress and inflammation and by inhibiting the digestive enzymes responsible for the hydrolysis of lipids and carbohydrates [6].

The Psychotria genus belongs to the family of Rubiaceae commonly used in traditional medicine for the treatment of several disease conditions. Phytochemical studies on the *Psychotria* genus revealed the presence of secondary metabolites such as alkaloids (indoles, monoterpene indoles, quinolines, and isoquinolines), flavonoids, terpenoids, and coumarins [7]. Amongst this genus, *Psychotria densinervia* is the most abundant and commonly found plant in the dense forest of Cameroon [8]. *Psychotria densinervia* is a large shrub measuring 3 m in height with a pale whitish-grey stem. Its petioles are pale green above and brown below. The leaves are smooth, leathery, and dark green. The flowers are yellow and the fruits are dull dark red to pale green. This plant is used by the local population in the treatment of malaria [8] and other complications related to cardiovascular diseases. It also has diuretic properties and facilitates digestion due to which it is referred to as a slimming plant in the locality of the Southern Region of Cameroon. However, studies related to the weight loss activity remain limited.

This study evaluated the antioxidant, anti-inflammatory, and digestive enzymes' inhibitory properties of hydro-ethanolic extracts of *Psychotria densinervia* leaves and bark.

## 2. Materials and Methods

### 2.1. Reagents

Ethanol, HCl, Mayer's reagent (potassium mercuric iodide solution), NaOH, hydrochloric acid, ferric chloride, potassium ferrocyanide, Fehling's solution, Folin-Ciocalteu, catechin, quercetin, 2,2 diphenyl-1-picrylhydrazyl radical (DPPH), 2,2′-azino-bis(3 ethylbenzothiazoline-6-sulfonic acid) (ABTS), sodium persulfate, phosphate buffer, trichloroacetic acid, bovine serum albumin (BSA), TPTZ (2,3,5-triphenyltetrazolium chloride) sodium diclofenac, phosphate buffer saline (PBS), trypsin, Tris/HCl buffer, casein, perchloric acid, porcine pancreatic lipase, pancreatic cholesterol esterase (PCase), pancreatic *α*-amylase, *α*-glucosidase, orlistat, p-nitro-phenyl butyrate, p-nitrophenyl-*α*-D-glucopyranoside, acarbose, and ascorbic acid were purchased from Sigma-Alrich Chemical Co.

### 2.2. Preparation of the Hydro-Ethanolic Leaf and Bark Extracts of *Psychotria densinervia*

**Figure 1:**
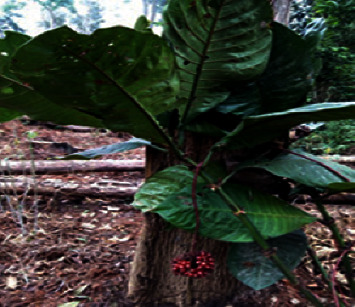
*Psychotria densinervia* (photo taken by Dr. Tsabang Nole, 2019).

The fresh leaves and bark of *P. densinervia* (Figure 1) were harvested in the Southern Region of Cameroon; the identification was done by Dr. Tsabang Nole at the National Herbarium of Yaoundé-Cameroon with the identification number No. 58226 HNC. Plant parts (leaves and bark) were washed with distilled water three times and shade-dried for two weeks at room temperature. Each dried material (546.2 g and 426.6 g for leaf and bark, respectively) was ground and macerated separately with a hydro-ethanolic solution (70% ethanol and 30% distilled water, v : v) at 35°C for three days. The extracts were then filtered and evaporated with the aid of a rotavapor, dried in an oven at 50°C, and labeled as PHELE (leaf extract) and PHEBE (bark extract). The PHELE and PHEBE were stored at −4°C for subsequent use.

### 2.3. Preliminary Phytochemical Screening

The presence of possible phytochemical components in the extracts was determined by color change due to the reaction between the principal reagents and specific bioactive components (phenolic compounds, flavonoids, alkaloids, tannins, coumarins, steroids, saponins, and terpenoids). The color intensity is determined by the abundance of a particular compound.

#### 2.3.1. Test for Phenolic Compounds

A few drops of 5% glacial acetic acid were added to 1 ml of each leaf and bark extract, followed by the addition of a few drops of 5% NaNO_2_ solution. The muddy brown color developed revealed the presence of phenols in the test samples [9].

#### 2.3.2. Test for Flavonoids

The extract (1 ml) was taken into a test tube and a few drops of diluted NaOH solution were added. An intense yellow color, which fades to colorless on the addition of a few drops of diluted acid, indicated the presence of flavonoids [10].

#### 2.3.3. Test for Alkaloids

Mayer's test was used for the determination of alkaloids [9]. According to this test procedure, 2 ml of concentrated HCl was added to 2 ml of the respective leaf and bark extract followed by the addition of a few drops of Mayer's reagent. The development of a white precipitate or green color confirmed the presence of alkaloids.

#### 2.3.4. Test for Tannins

A volume of 2 ml of 1N NaOH was added to 2 ml of each leaf and bark extract. The change from yellow to red color revealed the presence of tannins [9].

#### 2.3.5. Test for Coumarins

A volume of 1 ml of 10% NaOH solution was added to 1 ml of each leaf and bark extract. The formation of yellow color confirmed the existence of coumarins in the tested samples [9].

#### 2.3.6. Test for Saponins

According to Hossain et al. [10], 20 ml of distilled water was added to 2 ml of the extracts and shaken vigorously for 15 mins in a graduated cylinder. A layer of foam up to 1 cm or more in thickness confirmed the presence of saponins.

#### 2.3.7. Test for Terpenoids

An aliquot of 1 ml of 1% HCl was added to 2 ml of the extracts and left to stand for 5 h. Later on, 1 ml of Trim-Hill reagent was added and heated in a boiling water bath for 10 mins. The appearance of bluish-green color indicated the presence of terpenoids [9].

#### 2.3.8. Test for Steroids

The crude plant extracts (1 mg) were taken in a test tube and dissolved with chloroform (10 ml), and then, an equal volume of concentrated sulfuric acid was carefully added. The presence of steroids was detected by a red upper layer and yellow sulfuric acid layer.

### 2.4. Assessment of *In Vitro* Antioxidant Activity

#### 2.4.1. DPPH (2,2-Diphenyl-1-picrylhydrazyl) Free Radical Scavenging Method

The DPPH scavenging assay was conducted by the method of Sanchez-Moreno et al. [11] with some modifications. DPPH˙ (0.1 mM) stock solution was diluted in methanol, and the absorbance was adjusted to 1.5 at 517 nm wavelength. *P. densinervia* extracts (500 *μ*l) at different concentrations (15.62, 31.25, 62.5, and 250 *μ*g/ml) were mixed with 2000 *μ*l of DPPH solution and read with the aid of a spectrophotometer at 517 nm after 30 mins of incubation in the dark. Ascorbic acid was used as a standard, and the percentage of inhibition was calculated using the following formula:

(1) $$\begin{array}{c} \text{percentage of inhibition}=\left(\frac{\text{blank}-\text{test}}{\text{blank}}\right)\times 100, \end{array}$$

where test = absorbance of the sample (plant extract or ascorbic acid) and blank = absorbance of DPPH alone. The 50% inhibition concentration (IC_50_) was calculated from the regression equation.

#### 2.4.2. ABTS (2,2′-Azinobis(3-ethylbenzothiazoline 6-Sulfonic Acid)) Radical Scavenging Method

ABTS scavenging activity was evaluated by the discoloration of the cationic radical ABTS^*∗*^^+^, as per the technique described by Arnao et al. [12] with some modifications. Briefly, the ABTS reagent was obtained by mixing an ethanolic solution of ABTS (7.4 mM) and an aqueous solution of potassium persulfate (2.6 mM). After mixing, the solution was incubated in the dark for 24 hours. The ABTS solution obtained was diluted until an absorbance of 1.5 was obtained at 734 nm. Two hundred microliters of each extract or catechin at different concentrations (15.62, 31.25, 62.5, and 250 *μ*g/ml) was added to 1800 *μ*l of the ABTS solution prepared above. The mixture was incubated in the dark for 15 mins, and the absorbance was read with a spectrophotometer at 734 nm against the blank. Catechin was used as the standard. The percentage of inhibition was calculated using the following formula:

(2) $$\begin{array}{c} \text{percentage of inhibition}=\left(\frac{\text{blank}-\text{test}}{\text{blank}}\right)\times 100, \end{array}$$

where test = absorbance of sample (plant extract or catechin) and blank = absorbance of ABTS alone. The IC_50_ was calculated as in DPPH.

#### 2.4.3. Ferric Reducing Antioxidant Power (FRAP) Assay

The FRAP was determined according to the method of Benzie and Strain [13] with slight modifications. FRAP reagent was prepared by mixing 2500 *μ*l of TPTZ (2,3,5-triphenyltetrazolium chloride), 2500 *μ*l of FeCl_3_, and 2500 *μ*l of acetic acid/sodium acetate buffer. Then, 75 *μ*L of each extract (1000 *μ*g/ml) was added to 2000 *μ*l of the FRAP reagent. The mixture was incubated at 37°C for 30 mins, and the absorbance was read at 595 nm against the blank. A calibration curve was plotted from the results of a diluted series of catechin (7.81, 15.62, 31.25, 62.50, 125, and 250 *μ*g/ml) treated in the same manner as the extracts. The results were expressed as milligrams of catechin equivalent per gram of extract (mg CE/g of extract).

#### 2.4.4. Total Phenolic Content (TPC) Assay

The total phenolic content was determined according to the Folin-Ciocalteu (FC) method [14] with slight modifications. Briefly, 200 *μ*l of extract or standard (1000 *μ*g/ml) was mixed with 1000 *μ*l of Folin-Ciocalteu reagent diluted 10 times and the mixture was incubated for 4 mins. Then, 800 *μ*l of sodium carbonate solution (75000 *μ*g/ml) was added, and the mixture was incubated for 2 hours at room temperature. Then, the absorbance was read in a spectrophotometer at 765 nm against a blank. A calibration curve was plotted from the results of a dilution series of catechin (7.81, 15.62, 31.25, 62.5, 125, and 250 *μ*g/ml). The results were expressed as milligrams of catechin equivalent per gram of extract (mg CE/g of extract).

#### 2.4.5. Flavonoid Content Assay

The flavonoid content was determined following the method described by Jia et al. [15] with some modifications. Briefly, 500 *μ*l of extract (500 *μ*g/ml) was mixed with 150 *μ*l of 5% sodium nitrate and incubated for 5 mins at room temperature. Then, 150 *μ*l of 10% aluminum trichloride and 1 ml of 1 M sodium hydroxide were added to the mixture and the volume was made up to 5000 *μ*l with distilled water. The mixture was incubated for 10 mins, and the absorbance was read in a spectrophotometer at 510 nm against a blank. A standard curve was plotted from the results of a series of catechin dilutions (7.81, 15.62, 31.25, 62.50, 125, and 250 *μ*g/ml). The results were expressed as milligrams of catechin equivalent per gram of extract (mg CE/g of extract).

### 2.5. Assessment of *In Vitro* Anti-Inflammatory Activity of Extracts

#### 2.5.1. Inhibition of Bovine Serum Albumin (BSA) Denaturation

Inhibition of protein denaturation was evaluated using the method of Rahman et al. [16]. Nine hundred microliters (900 *μ*l) of 1% bovine serum albumin (BSA) was added to 100 *μ*l of the extracts at varying concentrations (62.5, 125, 250, 500, and 1000 *μ*g/ml). This was then allowed to stand at room temperature for 10 mins, followed by heating at 70°C for 15 mins. The reaction mixture was then allowed to cool at room temperature, and the absorbance was recorded at 660 nm. Diclofenac sodium was used as a positive control. The percentage inhibition of protein denaturation was calculated using the formula below:

(3) $$\begin{array}{c} \text{Percentage of inhibition}=\left(\frac{\text{blank}-\text{test}}{\text{blank}}\right)\times 100, \end{array}$$

where test = absorbance of sample (plant extract or diclofenac sodium) and blank = absorbance of enzyme + substrate.

The IC_50_ (the concentration causing 50% of inhibition) was obtained from the linear regression curve.

#### 2.5.2. Proteinase Inhibitory Action

The test was conducted in accordance with the method described by Gulnaz [17] with some modifications. The reaction mixture (2 ml) contained 60 *μ*g of trypsin, 1000 *μ*l of 20 mM Tris HCl buffer (pH 7.4), and 1 ml extract at different concentrations (62.5, 125, 250, 500, and 1000 *μ*g/ml). The mixture was incubated at 37°C for 5 mins and then 1 ml of 0.8% (w/v) casein was added. The mixture was incubated for an additional 20 mins at 37°C. Hence, 2 ml of 70% perchloric acid was added to stop the reaction. A cloudy suspension was centrifuged at 3000 rpm (revolutions per min) for 10 mins, and the absorbance of the supernatant (containing short chains of amino acids, dipeptides, and polypeptides) was read at 210 nm against Tris HCl buffer as blank. The percentage inhibition of proteinase activity was calculated as earlier mentioned above. The values of the IC_50_ were graphically obtained from the linear regression.

### 2.6. Inhibiting the Activity of Digestive Enzymes

#### 2.6.1. Pancreatic Lipase Inhibition Methods

The method described by Kim et al. [18] was used in this assay. Eighty microliters (80 *μ*l) of each extract at different concentrations (3.125, 6.25, 12.5, 25, 50, 100, and 200 *μ*g/ml) was mixed with 20 *μ*l of porcine pancreatic lipase (4 mg/ml) and 90 *μ*l of phosphate buffer. The mixture was then incubated at 37°C for 37 mins. The reaction was started by the addition of 10 *μ*l of *p*-nitrophenyl butyrate substrate (10 mM *p*-NPB) in dimethylformamide. After 30 mins of incubation at 37°C, the lipase inhibitory activity was determined by measuring the hydrolysis of *p*-NPB to *p*-nitrophenol at 405 nm using an ELISA microplate reader (BK-EL10 C). Orlistat was used as the standard. The percentage of inhibition was calculated using the following formula:

(4) $$\begin{array}{c} \text{percentage of inhibition}=\left(\frac{\text{blank}-\text{test}}{\text{blank}}\right)\times 100, \end{array}$$

where test = absorbance of sample (plant extract or orlistat) and blank = absorbance of enzyme + substrate.

Antilipase activity is given as IC_50_ values (the concentrations that inhibited the hydrolysis of *p*-NPB to *p*-nitrophenol by 50%).

#### 2.6.2. Cholesterol Esterase Inhibition Method

The inhibition of pancreatic cholesterol esterase was carried out according to the method described by Adisakwattana et al. [19]. Briefly, 50 *μ*l of the extracts at different concentrations (3.12, 6.25, 12.5, 25, 50, 100, and 200 *μ*g/ml) was added to 50 *μ*l of 5.16 mM taurocholic acid and 50 *μ*l of an aqueous solution of cholesterol esterase and incubated for 10 mins at 25°C. Then, 50 *μ*l of *p*-nitrophenyl butyrate substrate (0.2 mM) was added to the mixture and incubated for 5 mins at 25°C. Cholesterol esterase inhibitory activity was determined by measuring the hydrolysis of *p*-NPB to *p*-nitrophenol at 405 nm using an ELISA microplate reader (BK-EL10 C). Orlistat was used as the standard. The percentage of inhibition was calculated using the following formula:

(5) $$\begin{array}{c} \text{percentage of inhibition}=\left(\frac{\text{blank}-\text{test}}{\text{blank}}\right)\times 100, \end{array}$$

where test = absorbance of sample (plant extract or orlistat) and blank = absorbance of enzyme + substrate.

Anticholesterol esterase activity is given as IC_50_ values (the concentrations that inhibited 50% of the hydrolysis of *p*NPB to *p*-nitrophenol).

#### 2.6.3. Alpha-Amylase Inhibition Assay

The method earlier described by Ademiluyi and Oboh [20] was used in this assay. Fifty microliters (50 *μ*l) of extract at different concentrations (3.12, 6.25, 12.5, 25, 50, 100, and 200 *μ*g/ml) was mixed with 10 *μ*l of *α*-amylase solution (500 *μ*g/ml) and incubated for 10 mins at 37°C. Then, 40 *μ*l of starch solution (0.25%) was added to the mixture and incubated for 30 mins at 37°C. Then, 20 *μ*l of 1M HCl was added to stop the enzyme reaction, and 80 *μ*l of Lugol was added to reveal the presence of starch. The intensity of the blue color was proportional to the amount of starch remaining. Acarbose was used as the positive control. The absorbance was read at 620 nm using an ELISA microplate reader (BK-EL10 C). The percentage of inhibition was calculated using the following formula:

(6) $$\begin{array}{c} \text{percentage of inhibition}=\left(\frac{\text{blank}-\text{test}}{\text{blank}}\right)\times 100, \end{array}$$

where test = absorbance of sample (plant extract or acarbose) and blank = absorbance of enzyme + substrate.

#### 2.6.4. Pancreatic Alpha-Glucosidase Inhibitory Activity

The effect of the plant extracts on alpha-glucosidase activity was determined according to the chromogenic method described by Mumtaz et al. [21] with slight modifications. Eighty microliters (80 *μ*l) of extract or acarbose (standard) at different concentrations (3.12, 6.25, 12.5, 25, 50, 100, and 200 *μ*g/ml) was added to 10 *μ*l of alpha-glucosidase 1 U/ml and incubated for 10 mins at 37°C. Then, 10 *μ*l of *p*-nitrophenyl-*α*-D-glucopyranoside was also added to the mixture and incubated at 37°C for 30 mins. Finally, 100 *μ*l of 0.2 M sodium carbonate was added to stop the reaction, and the absorbance was read at 405 nm using an ELISA microplate reader (BK-EL10 C). Percentage inhibition was calculated using the following formula:

(7) $$\begin{array}{c} \text{percentage of inhibition}\left(\%\right)=\left(\frac{\text{blank}-\text{test}}{\text{blank}}\right)\times 100, \end{array}$$

where test = absorbance of sample (plant extract or acarbose) and blank = absorbance of enzyme + substrate.

### 2.7. Statistical Analysis

All the results (in triplicate) were expressed as mean ± standard deviation (SD) using Microsoft Excel 2016. Statistical analysis for group comparison was carried out by using analysis of variance (ANOVA). Significant differences between groups were determined by Dunnett's multiple comparison test at *p* < 0.001. The statistical software used was GraphPad prism 5.

## 3. Results

### 3.1. Preliminary Phytochemical Screening

The phytochemical screening of the extracts of *P. densinervia* revealed the presence of secondary metabolites such as alkaloids, phenolic compounds, flavonoids, coumarins, steroids saponins, terpenoids, and tannins, presented in Table 1. The polyphenols, flavonoids, terpenoids, and steroids were more abundant in the *P. densinervia* leaf extract than the bark extract, while the composition of alkaloids, coumarins, saponins, and tannins was similar in both the extracts.

### 3.2. Antioxidant Properties

#### 3.2.1. DPPH (2,2-Dipheny l-1-picrylhydrazyl) Free Radical Scavenging

**Figure 2:**
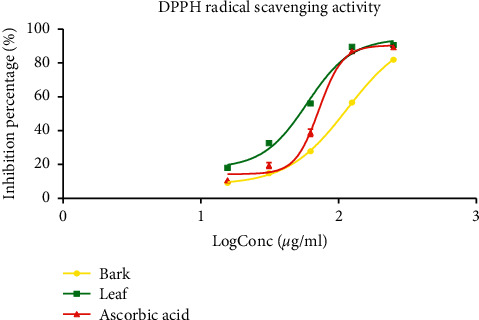
DPPH scavenging activity of the hydro-ethanolic leaf and bark extracts of *Psychotria densinervia* and ascorbic acid. Values are expressed as mean ± SD (*n* = 3).

The percentage of DPPH radical scavenging activity of the extracts was dose-dependent (Figure 2). The curve observed for both the extract and the standard were sigmoidal turning toward saturation at optimal concentration. At 250 *μ*g/ml concentration, all extracts showed inhibition percentages greater than 80%. The IC_50_ of DPPH radical scavenging activity is presented in Table 2. The leaf extract showed better DPPH radical scavenging activity with a significantly low IC_50_ value (59.09 ± 5.97 *μ*g/ml, *p* < 0.001) compared to ascorbic acid (IC_50_ = 71.78 ± 6.37 *μ*g/ml) and bark extract (IC_50_ = 115.40 ± 1.21 *μ*g/ml). The lower the IC_50_ is, the better the radical scavenging activity.

#### 3.2.2. ABTS Free Radical Scavenging

**Figure 3:**
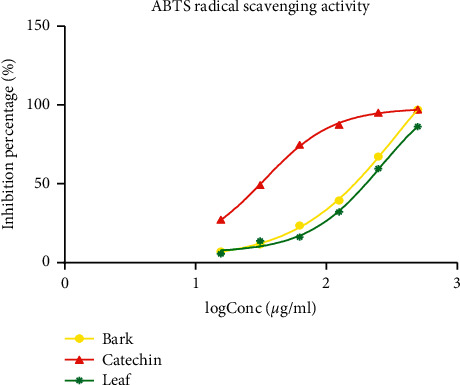
ABTS scavenging activity of the hydro-ethanolic leaf and bark extracts of *Psychotria densinervia* and ascorbic acid. Values are mean ± SD (*n* = 3).

Figure 3 presents the ABTS free radical scavenging activity of the plant extracts. At a concentration of 500 *μ*g/ml, all extracts exhibited a percentage of radical scavenging activity greater than 80%. However, catechin had the highest inhibition percentage compared to both the plant extracts. The IC_50_ of the extract scavenging the ABTS radical activity is presented in Table 2. It was observed that catechin exhibited the lowest IC_50_ indicating the highest antioxidant activity (IC_50_ = 33.48 ± 2.02 *μ*g/ml) followed by the leaf extract (262.4 ± 4.46 *μ*g/ml) and then the bark extract (354.2 ± 1.97 *μ*g/ml).

#### 3.2.3. Ferric Reducing Antioxidant Potential (FRAP)

Ferric reducing antioxidant power of the extracts is presented in Table 2. The leaf extract exhibited a significantly higher FRAP activity (341.73 ± 2.17 mg CE/g of extract, *p* < 0.001) than the bark extract (150.30 ± 0.32 mg CE/g of extract).

#### 3.2.4. Determination of Polyphenol and Flavonoid Content

The polyphenol and flavonoid content in the dried bark and dried leaves was determined in milligrams equivalent catechin per gram of *P. densinervia* extracts (Table 3). The leaf extract significantly showed a higher polyphenol content (270.05 ± 7.53 mg CE/*g*g of extract, *p* < 0.001) compared to the bark extract (138.89 ± 0.91 mg CE/*g* of extract). In addition, it was observed that the flavonoid content of the leaf extract (23.43 ± 0.03 mg CE/*g* of extract) was significantly (*p* < 0.01) higher than that of the bark extract (20.06 ± 0.03 mg CE/*g* of extract).

### 3.3. Anti-Inflammatory Assays

#### 3.3.1. Protein Denaturation Inhibition Test

**Figure 4:**
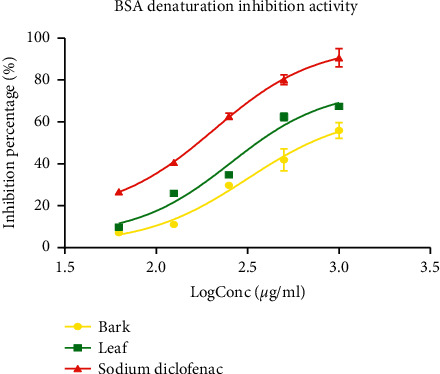
Heat-induced BSA denaturation inhibitory activity of the hydro-ethanolic leaf and bark extracts of *Psychotria densinervia* and sodium diclofenac. Values are expressed as mean ± SD (*n* = 3).

The data obtained for the anti-inflammatory property of *P. densinervia* revealed that protein denaturation inhibition was dose-dependent (Figure 4). The highest inhibition percentage of protein denaturation was obtained by sodium diclofenac, the standard drug.  Table 4 presents the IC_50_ values in (*μ*g/ml) of the extracts and sodium diclofenac against protein denaturation. The leaf extract showed a significant (*p* < 0.001) lower IC_50_ with a value of 257.0 ± 7.51 *μ*g/ml compared to the bark extract with the IC_50_ value as 316.1 ± 6.02 *μ*g/ml. Furthermore, IC_50_ of the leaf extract was significantly (*p* < 0.001) higher than the sodium diclofenac IC_50,_ the standard drug (207.6 ± 3.94 *μ*g/ml).

#### 3.3.2. Antiproteinase Activity

**Figure 5:**
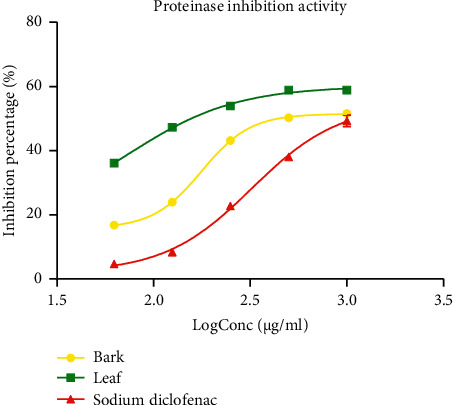
Antiproteinase activity of the hydro-ethanolic leaf and bark extracts of *Psychotria densinervia* and sodium diclofenac. Values are expressed as mean ± SD (*n* = 3).

The antiproteinase activity of the extracts and the reference standard (sodium diclofenac) are presented in Figure 5. The antiproteinase action of both *P. densinervia* extracts and sodium diclofenac were concentration-dependent. The inhibition percentage of the proteinase activity of the leaf extract was higher than that of the bark extract, which was in turn higher than that of sodium diclofenac. Both extracts of *P. densinervia* showed good antiproteinase activity compared to sodium diclofenac. The IC_50_ of the leaf extract (74.37 ± 1.10 *μ*g/ml) was significantly (*p* < 0.001) lower than the bark extract (IC_50_ = 177.6 ± 0.50 *μ*g/ml), which was also significantly lower than the sodium diclofenac (IC_50_ = 322.8 ± 2.75 *μ*g/ml) (Table 4). This indicates the effectiveness of the extract in inhibiting protein digestive enzymes.

### 3.4. Digestive Enzymes' Inhibition

#### 3.4.1. Inhibition of Pancreatic Lipase

**Figure 6:**
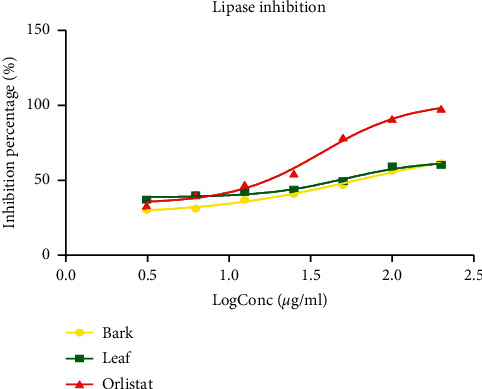
Lipase inhibitory activity of the hydro-ethanolic leaf and bark extract of *Psychotria densinervia* and orlistat. Values are expressed as mean ± SD (*n* = 3).

Figure 6 presents the lipase inhibitory activity of extracts and the standard. Data presented showed that both the extract and reference standard (orlistat) inhibited the lipase activity in a concentration-dependent manner. The inhibition percentage of lipase by *P. densinervia* hydro-ethanolic leaf and bark extracts (61.49 ± 0.36% and 60.32 ± 0.54 respectively) were lower than that of the standard orlistat (97.83 ± 0.34%) at a concentration of 200 *μ*g/ml. The best inhibiting activity of pancreatic lipase was found with orlistat, the reference standard, where the IC_50_ value was 37.11 ± 4.39 *μ*g/ml, which was significantly lower (*p* < 0.001) compared to leaf and bark extracts with an IC_50_, respectively ,of 50.57 ± 2.89 *μ*g/ml and 62.88 ± 1.74 *μ*g/ml, respectively (Table 5).

#### 3.4.2. Pancreatic Cholesterol Esterase Inhibition

**Figure 7:**
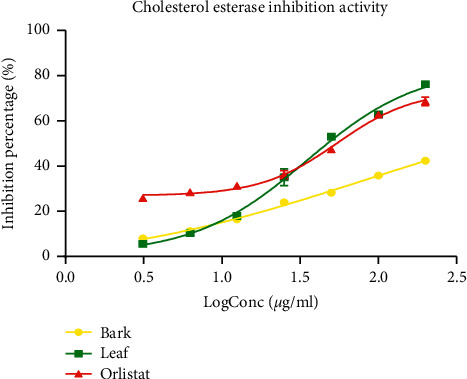
Cholesterol esterase inhibitory activity of the hydro-ethanolic leaf and bark extracts of *Psychotria densinervia* and orlistat. Values are expressed as mean ± SD (*n* = 3).

The effect of both extracts and reference drug (orlistat) on cholesterol esterase activity is presented in Figure 7. It is observed that all the three tested samples had a significant pancreatic cholesterol esterase inhibition activity, which was concentration-dependent. The bark extract of *P. densinervia* had the lowest inhibitory effect on cholesterol esterase activity. At the smallest concentration, orlistat had a better inhibition percentage right to the log concentration of 1.5 *μ*g/ml, where there was an intercept with the *P. densinervia* leaf extract. At log concentration above 1.5 *μ*g/ml, the leaf extract of *P. densinervia* presented a higher percentage of inhibition activity compared to orlistat. The calculated IC_50_ for *P. densinervia* extracts and orlistat are presented in Table 5. The IC_50_ of the leaf extract (34.75 ± 3.87 *μ*g/ml) was significantly (*p* < 0.001) lower than that of the reference molecule orlistat (54.61 ± 2.56 *μ*g/ml) and bark extract (IC_50_ = 80.14 ± 1.71 *μ*g/ml).

#### 3.4.3. Inhibition of Pancreatic *α*-Amylase

**Figure 8:**
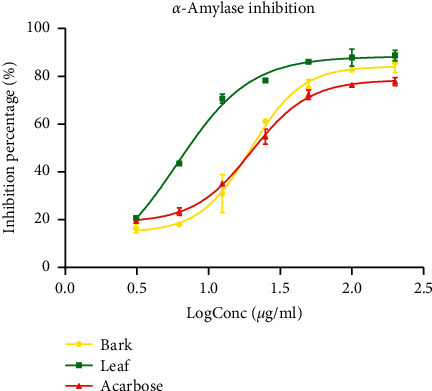
*α*-Amylase inhibitory activity of the hydro-ethanolic leaf and bark extract of *Psychotria densinervia* and acarbose. Values are expressed as mean ± SD (*n* = 3).

The inhibitory activity of *P. densinervia* hydro-ethanolic extracts and acarbose on pancreatic *α*-amylase activity showed a concentration-response effect (Figure 8). All the three tested samples presented a sigmoidal curve reaching saturation at the log concentration of 1.75 *μ*g/ml. The results of the leaf extract showed the highest percentage inhibition of pancreatic *α*-amylase activity compared to bark extract and acarbose. Considering the IC_50_ values, the leaf extract had a value (IC_50_ = 6.06 ± 4.05 *μ*g/ml), which was significantly lower (*p* < 0.001) than that of the bark extract (IC_50_ = 19.69 ± 6.27 *μ*g/ml) and the reference drug acarbose (IC_50_ = 20.01 ± 2.84 *μ*g/ml). This makes the leaf extract more potent in the inhibition of *α*-amylase activity. The bark extract showed an inhibitory activity similar to acarbose (Table 5).

#### 3.4.4. Inhibition of Pancreatic *α*-Glucosidase

**Figure 9:**
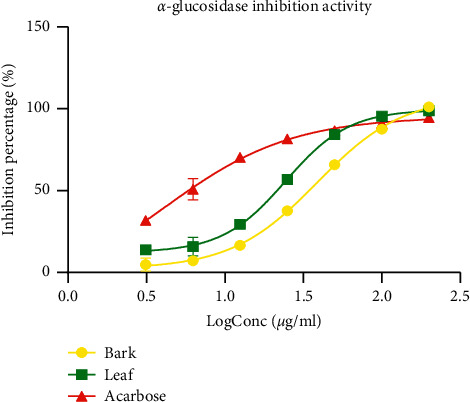
*α*-Glucosidase inhibitory activity of the hydro-ethanolic leaf and bark extracts of *Psychotria densinervia* and acarbose. Values are expressed as mean ± SD (*n* = 3).

The results of the percentage inhibition of *α*-glucosidase activity of *P. densinervia* hydroethanolic extracts and acarbose also showed a concentration-response effect (Figure 9). At a log concentration of 0.5 to 1.75 *μ*g/ml, the reference drug acarbose showed a higher percentage inhibition of *α*-glucosidase than *P. densinervia* hydroethanolic extracts. At the log concentration of 2 to 2.5 *μ*g/ml, the leaf extract had a higher percentage inhibition effect on *α*-glucosidase than acarbose. When calculated, the IC_50_ of acarbose was significantly lower (4.11 ± 3.47 *μ*g/ml, *p* < 0.001) compared to the leaf extract (IC_50_ = 24.41 ± 2.84 *μ*g/ml) and bark extract (IC_50_ = 38.81 ± 2.46 *μ*g/ml) (Table 5). This makes acarbose a better inhibitor of *α*-glucosidase than the plant extracts. However, the leaf extract has shown greater inhibitory activity than the bark extract.

## 4. Discussion

Obesity is defined as a chronic state of oxidative stress and inflammation, even in the absence of other risk factors, indicating that these metabolic mechanisms (oxidative stress and inflammation) are present and could contribute to the development of the metabolic syndrome [22]. Metabolic syndrome is responsible for the development of illnesses such as type 2 diabetes, cardiovascular disease, arthritis, hypertension, cardiac arrest, and certain cancers [23, 24].

Phytochemical screening (Table 1) of *P. densinervia* extracts revealed the presence of secondary metabolites such as alkaloids, phenolic compounds, flavonoids, coumarins, steroids saponins, terpenoids, and tannins. Alkaloids have earlier been associated with reduction in the expression of adipocyte marker genes, which enhances binding proteins and proliferator-activated receptor, thereby inhibiting adipogenesis, leading to antiobesity activities [25, 26]. On the other hand, flavonoids and phenols have been reported to exert an antioxidant activity, which may be important in modulating obesity-related oxidative stress in the body [25, 26]. Coumarins possess antiobesity activities mainly by inhibiting lipogenesis in adipocytes [27]. Steroids have been known to contribute to cholesterol and low-density lipoprotein reduction in serum, and compete with cholesterol for micelle formation in the intestinal lumen [26, 28]. Saponins and flavonoids are known to decrease the level of triglycerides and total cholesterol by the formation of large micelles excreted in bile. Also, they decrease the absorption of cholesterol in the intestines and serum levels of low-density lipoprotein cholesterol, playing a role in weight loss [26, 29]. Terpenoids are involved in hypolipidemic activity by inhibiting pancreatic lipase [30]. Tannins have antioxidant and antilipase activities [31]. Both leaf and bark extracts of *P. densinervia* contain these bioactive components and may be responsible in one way or the other for the biological activities of the plant. The leaf extract of *P. densinervia* presented denser coloration in phytochemistry studies indicating the abundance of phytochemical constituents responsible for the antiobesity activity [32].

In the DPPH and ABTS radical scavenging activities, the hydro-ethanolic leaf extract of *P. densinervia* showed a higher free-radical scavenging activity compared to the hydro-ethanolic bark extract of *P. densinervia*. This is correlated with the high total polyphenols and flavonoids content, which are higher than that of the bark extract. In the ABTS test, it appears that catechin exhibited the highest ABTS scavenging activity than the leaf extract. However, for the DPPH and ABTS tests at the concentration of 250 *μ*g/ml, both leaf and bark extracts exhibited an inhibition percentage greater than 80%. It has been reported that the production of free radicals responsible for oxidative stress is equally increased in adipose tissues and liver of mice fed with a high-fat diet [33]. Therefore, the free radical scavenging capacities exhibited in this study by the leaf and bark extract might have occurred through the transfer of electrons and hydrogen atoms in the DPPH test and through electron donation in the ABTS test, which is important for the prevention of oxidative stress development.

Phytochemical compounds often possess ferric reducing capacity as electron donors, thus reducing oxidized intermediates such as those of lipid peroxidation processes [34]. In this study, the hydro-ethanolic leaf extract of *P. densinervia* exhibited a higher ferric reducing antioxidant power than the hydro-ethanolic bark extract. The antioxidant potential of the leaf extract can play a role in the prevention of protein denaturation, lipid peroxidation, and the disruption of membrane fluidity implicated in oxidative stress [34]. The marked electron donation ability of the leaf extract could be attributed to its polyphenolic and flavonoid content, which can transfer electrons to neutralize free radicals, chelate metal catalysts, and activate antioxidant enzymes [34].

Systemic inflammation-associated obesity is characterized by increased circulating concentrations of proinflammatory cytokines and chemokines, and activation of pathways that regulate inflammation. A protein denaturation assay was used in this study as an evidence for the membrane-stabilizing properties. Proteins are denatured when they lose their secondary and tertiary structures through the application of external stress or compounds such as heat, strong acids, or bases. The denaturation of tissue proteins leads to the production of autoantigens [35, 36]. Therefore, any agent that can prevent protein denaturation is worth considering for anti-inflammatory drug development. Most nonsteroidal anti-inflammatory drugs (NSAIDs) are known to possess the intrinsic ability to stabilize or prevent heat-treated albumin denaturation at physiological pH 6.2–6.5 [35, 37]. Also, it has been reported that the administration of NSAIDs to overweight patients may improve the loss in their body weight [38]. In the present investigation, the leaf extract exhibited a higher protein denaturation inhibition than the bark extract. The activity of the leaf extract was lower than the sodium diclofenac, which implied that this extract could be used as an alternative to synthetic NSAIDs.

Leukocytes, in their lysosomal granules, carry many serine proteinases [39, 40]. Thus, during inflammation, as part of their defensive roles, leukocytes release their lysosomal enzymes, including proteinases, causing further tissue damage and subsequent inflammation [39, 41]. Damage to cell membranes will further make the cell more susceptible to secondary damage by free radical-induced lipid peroxidation [39, 42], which may lead to atherosclerosis. In this study, the hydro-ethanolic leaf extract showed higher antiproteinase activity than the sodium diclofenac, which was higher than the hydro-ethanolic bark extract. It might be suggested that *P. densinervia* extracts and especially the leaf extract might inhibit the release of the lysosomal constituents of leukocytes at the site of inflammation.

The development of obesity is closely related to the metabolism of body fat. Exogenous fat cannot be directly used by the human body without being hydrolyzed for absorption [43]. Pancreatic lipase hydrolyzes triacylglycerol into free fatty acids in the intestine for absorption [43]. From there, the monoglycerides and free fatty acids are subsequently moved to enterocytes, cells lining the intestines, and then absorbed [43, 44]. In fact, the inhibition of pancreatic lipase activity is expected to limit dietary fat absorption, resulting in delayed triglyceride digestion. The hydro-ethanolic leaf extract showed a significantly higher activity than the bark extract. The hydroxyl groups of phenolic compounds present in extracts are reported to form hydrophobic interactions with amino acid residues of pancreatic lipase, which lead to inhibition [45]. Dietary cholesterol consists of both free and esterified cholesterol. Esterified cholesterols are hydrolyzed by pancreatic cholesterol esterase to release free cholesterol in the small intestines [46]. Moreover, it plays an important role in regulating the incorporation of cholesterol into mixed micelles [47] and its transportation into the blood plasma. Therefore, the inhibition of cholesterol esterase is crucial to restrict and delay the absorption of dietary cholesterol in the small intestine. Lowering of cholesterol absorption by inhibiting the cholesterol esterase is a good strategy for the management of hyperlipidemia and obesity [48, 49]. The hydro-ethanolic leaf extract of *P. densinervia* showed a significantly higher inhibition than the reference orlistat and bark extract *P. densinervia*. The mechanism of cholesterol esterase inhibition may be due to the interaction of phenolic compounds contained in extracts with the potent cholesterol esterase inhibitory sites, especially the interaction with the catalytic triad and oxyanion hole residues [50].

Alpha-amylase and *α*-glucosidase are two enzymes involved in the hydrolysis of polysaccharides and disaccharides into simple sugars [51]. Inhibition of these enzymes impairs the digestion of carbohydrates and limits their absorption into the bloodstream [51]. This inhibition could be a way of fighting obesity due to hepatic lipogenesis. Inhibition of *α*-glycosidase prolongs gastric emptying, leading to satiety and weight loss, effects of which may be useful in the treatment of obesity [52]. *P. densinervia* leaf extract showed a significantly high inhibition of *α*-amylase than the reference acarbose and the bark extract. In the *α*-glycosidase test, acarbose showed significant high activity. The leaf extract exhibited a higher activity than the bark extract. These activities may be due to the presence of different bioactive compounds contained in the extracts.

## 5. Conclusion

This study examines the *in vitro* antioxidant, anti-inflammatory, and digestive enzymes inhibition activities of hydro-ethanolic leaf and bark extracts of *Psychotria densinervia*. The results showed that the hydro-ethanolic leaf and bark extracts of *Psychotria densinervia* contained bioactive compounds such as alkaloids, phenolic compounds, flavonoids, coumarins, steroids saponins, terpenoids, and tannins. The hydro-ethanolic leaf extract exhibited a higher total phenolic and flavonoids content than the bark extract. The hydro-ethanolic leaf and bark extracts showed good antioxidant, anti-inflammatory activities, as well as good enzyme digestion inhibition activities. The leaf extract was more effective than the bark extract in all activities. Therefore, *Psychotria densinervia* hydro-ethanolic leaf extract could be used for the management of obesity. However, several mechanisms of action of the plant need to be elucidated.

## Figures and Tables

**Table 1:** Phytochemical screening of *P. densinervia* extracts.

Compounds	PHEBE	PHELE
Polyphenols	+	++
Flavonoids	+	++
Terpenoids	+	++
Coumarins	++	++
Steroids	+	++
Tannins	++	++
Alkaloids	+	+
Saponins	T	T

(++): strongly positive test; (+): positive test; (-): negative test; T: trace.

**Table 2:** IC_50_ and correlation coefficient (*r*) of the hydro-ethanolic leaf and bark extracts in DPPH, ABTS assay, and the ferric reducing power activity in the FRAP assay.

Test	Parameters	Inhibitors		
		Standard	Bark	Leaf
DPPH	IC_50_ (*μ*g/ml)	71.98 ± 1.21	115.40 ± 5.97^*∗∗∗*^	59.09 ± 6.37^*∗∗∗*^^†^
	R^2^	0.9910	0.9995	0.9898

ABTS	IC_50_ (*μ*g/ml)	33.48 ± 2.02	354.2 ± 1.97^*∗∗∗*^	262.4 ± 4.46^*∗∗∗*^^†^
	R^2^	0.9984	0.9986	0.9937

FRAP	mg CE/g of extract		150.30 ± 0.32	341.73 ± 21.70^†^

Data are expressed as mean ± SD, *n* = 3; significantly different at ^*∗∗∗*^*p* < 0.001 when compared to standards (ascorbic acid in the DPPH test and catechin in the ABTS test); mg CE/g of extract: milligrams of catechin equivalent per gram of extract. ^†^*p* < 0.001 compared to bark extract.

**Table 3:** Total phenolic and flavonoids contents of the hydro-ethanolic leaf and bark extracts of *Psychotria densinervia*.

Extracts	Total phenolic content (mg CE/g of extract)	Total flavonoid content (mg CE/g of extract)
Leaf	270.05 ± 7.53^*∗∗∗*^	23.43 ± 0.03^*∗∗*^
Bark	138.89 ± 0.91	20 .06 ± 0.03

Data are expressed as mean ± SD; *n* = 3; mg CE/g of extract: milligram catechin equivalent per gram of extract. Significantly different at ^*∗∗∗*^*p* < 0.001 and ^*∗∗*^*p* < 0.01.

**Table 4:** IC_50_ and correlation coefficient (*r*) of hydro-ethanolic leaf and bark extracts in BSA denaturation and antiproteinase assay compared to sodium diclofenac.

Test	Parameters	Inhibitors		
		Sodium diclofenac	Bark	Leaf
BSA denaturation	IC_50_ (*μ*g/ml)	207.6 ± 3.94	316.1 ± 6.02^*∗∗∗*^	257.0 ± 7.51^*∗∗∗*^^†^
	R^2^	0.9933	0.9741	0.9711

Antiproteinase action	IC_50_ (*μ*g/ml)	322.8 ± 2.75	177.6 ± 0.50^*∗∗∗*^	74.37 ± 1.10^*∗∗∗*^^†^
	R^2^	0.9936	0.9997	0.9959

Data are expressed as mean ± SD; *n* = 3; significantly different at ^*∗∗∗*^*p* < 0.001 when compared to sodium diclofenac. ^†^*p* < 0.01 compared to bark extract.

**Table 5:** IC_50_ and correlation coefficient (*r*) of hydro-ethanolic leaf and bark extracts in lipase, cholesterol esterase, *α*-amylase, and *α*-glucosidase inhibition activity compared to standards.

Enzymes	Parameters	Inhibitors		
		Standards	Bark	Leaf
Lipase	IC_50_ (*μ*g/ml)	37.11 ± 4.39	62.88 ± 1.74^*∗∗∗*^	50.57 ± 2.89^*∗∗∗*^^†^
	R^2^	0.9908	0.9931	0.9641

Cholesterol esterase	IC_50_ (*μ*g/ml)	54.61 ± 2.56	80.14 ± 1.71^*∗∗∗*^	34.75 ± 3.87^*∗∗∗*^^†^
	R^2^	0.9938	0.9949	0.9945

*α*-Amylase	IC_50_ (*μ*g/ml)	20.01 ± 2.84	19.69 ± 6.27	6.06 ± 4.05^*∗∗∗*^^†^
	R^2^	0.9967	0.9903	0.9953

*α*-Glucosidase	IC_50_ (*μ*g/ml)	4.11 ± 3.47	38.81 ± 2.46^*∗∗∗*^	24.41 ± 2.84^*∗∗∗*^^†^
	R^2^	0.9898	0.9979	0.9949

Data are expressed as mean ± SD, *n* = 3; significantly different at ^*∗∗∗*^*p* < 0.001 when compared to standards (orlistat) in lipase and cholesterol esterase inhibition tests; acarbose in *α*-amylase and *α*-glucosidase inhibition test). ^†^*p* < 0.001 compared to bark extract.

## Data Availability

All data used in this study are presented as results.
